# The effects of physical activity on glutamate neurotransmission in neuropsychiatric disorders

**DOI:** 10.3389/fspor.2023.1147384

**Published:** 2023-03-06

**Authors:** Richard Baskerville, Thomas McGrath, Lindy Castell

**Affiliations:** ^1^ Faculty of Health and Life Sciences, Oxford Brookes University, Oxford, United Kingdom; ^2^ Green Templeton College, Oxford, United Kingdom

**Keywords:** exercise, neuroimmunology, depression, glutamate, inflammation, stress

## Abstract

Physical activity (PA) is an effective way of increasing cognitive and emotional health and counteracting many psychiatric conditions. Numerous neurobiological models for depression have emerged in the past 30 years but many struggle to incorporate the effects of exercise. The hippocampus and pre-frontal cortex (PFC) containing predominantly glutamate neurotransmission, are the centres of changes seen in depression. There is therefore increasing interest in glutamatergic systems which offers new paradigms of understanding mechanisms connecting physical activity, stress, inflammation and depression, not explained by the serotonin theories of depression. Similar hippocampal glutamate dysfunction is observed in many other neuropsychiatric conditions. Excitatory glutamate neurones have high functionality, but also high ATP requirements and are therefore vulnerable to glucocorticoid or pro-inflammatory stress that causes mitochondrial dysfunction, with synaptic loss, culminating in depressed mood and cognition. Exercise improves mitochondrial function, angiogenesis and synaptogenesis. Within the glutamate hypothesis of depression, the mechanisms of stress and inflammation have been extensively researched, but PA as a mitigator is less understood. This review examines the glutamatergic mechanisms underlying depression and the evidence of physical activity interventions within this framework. A dynamic glutamate-based homeostatic model is suggested whereby stress, neuroinflammation and PA form counterbalancing influences on hippocampal cell functionality, which manifests as depression and other neuropsychiatric conditions when homeostasis is disrupted.

## Introduction

Depression is defined by diagnostic criteria from the Diagnostic and Statistical Manual 5 (DSM5). including depressed mood or anhedonia (loss of interest or pleasure), with additional symptoms including appetite or weight changes, difficulty sleeping, diminished ability to think or concentrate, fatigue or loss of energy, feelings of worthlessness, or excessive guilt and suicidality (1). The heterogeneity of clinical presentations, presence of comorbid conditions and psychosocial confounding variables is challenging for the reproducibility of studies of the underlying mechanisms.

Major depressive disorder (MDD) is a leading cause of global disease burden that affects over 300 million persons worldwide (2). This burden is high across the entire lifespan, genders, global distributions and the incidence is rising. The total estimated number of people living with depression worldwide increased by 18.4% between 2005 and 2015 to 322 million equating to 4.4% of the world’s population, with a lifetime risk of 15%–18%. In terms of disability, depression ranked third in the causes of years lived with disabilities (3). Importantly, no reduction in the global prevalence or burden has been detected for depression or anxiety since 1990, despite compelling evidence of interventions that reduce their impact (4). The high costs to individuals and society demand efficacious treatments, yet 30% of patients fail to respond to current serotoninergic-based pharmacotherapeutics, while 70% do not achieve complete remission (5). Outcomes for Major Depressive Disorder (MDD) are associated with a reduced life expectancy of 10–20 years, despite active treatment. This is mostly due to cardiometabolic disease related to chronic treatments and physical inactivity (6). The high levels of treatment resistance, undesirable side effects, and the high economic and social cost to society have prompted a diversification of the search for effective treatment options (7).

Physical activity (PA) in its many forms has, since antiquity, been associated with antidepressant effects although the neurobiological mechanisms have remained elusive due to the heterogeneity of clinical depression and difficulty of studying neurochemistry *in vivo*, large differences in individual responses and poor uptake of sustained exercise interventions (8).

Volumetric magnetic resonance (MR) imaging of depressed patients has established for some time that, more than other brain areas, the hippocampus shows volume reduction in chronic psychiatric conditions which is partially restored by regular exercise (9, 10). However, previous models of depression, such as the monoamine theory of serotonin and noradrenaline, fail to provide a mechanistic framework for these effects, leading to a historical lack of research into exercise as a treatment.

There have been many neurobiological theories of depression, with many overlapping themes. The theories of monoamines, hypothalamic pituitary adrenal (HPA) axis hyperactivity, reduced neuroplasticity, hypoGABA-ergic theory and neuroinflammation are all validated hypotheses (11–15). Glutamate neurotransmission and associated mitochondrial function within the hippocampus, underpin and therefore unify many of these related concepts. However advances since the 2000s in knowledge of glutamate neurotransmission in the limbic and prefrontal cortex, have generated an overarching glutamate hypothesis of depression, which has a wide evidence base and increasing clinical acceptance (16). Importantly, a glutamate-based model of depression provides mechanistic explanations for how physical activity impacts on mental health. Therefore, the aim of this review is to provide an integrative view of neuroplasticity, glutamatergic and inflammatory theories of depression, centred on mitochondrial dysfunction in the hippocampus and to describe the integral role of PA within this schema. As depression is by far the most researched field for the effects of PA, we discuss the mechanisms for a model in relation to depression and then extend to other disorders. Given the rising societal prevalences of mental health disorders, social isolation, stress and sedentariness, the need to research the precise effects of exercise is more important than ever. Psychiatric conditions where PA has an evidence base and areas where PA remains under-researched are also discussed.

## Glutamatergic model of depression

The glutamate hypothesis of depression emerged in the 1990s, when antagonists of the N-methyl-D-aspartate (NMDA) receptor, an ionotropic glutamate receptor, produced antidepressant-like effects in mice (17). In a seminal human trial by Berman in 2000, ketamine (a long-standing anaesthetic agent and glutamate NMDA receptor antagonist) demonstrated a rapid-onset and prolonged mood improvement in MDD, including in cases of treatment-resistant depression (TRD) (18). Further proof of concept emerged when Magnetic Resonance Spectroscopy (MRS) scans revealed decreased levels of glutamatergic metabolites in the medial frontal cortex in patients with depression (19) and MR imaging showed volume reductions in MDD patients in the hippocampus which is a glutamate neurotransmission predominant area (20).

This prompted research into glutamate receptor subtypes and ligands as possible therapeutic targets and agents respectively. There are two classes of receptors that exhibit functional differences (21). Inotropic GluRs (iGluRs) are ligand-gated ion channels for fast signal transmission. Metabotropic receptors, GluRs (mGluRs) are G protein-coupled receptors (GPCRs) which control cellular processes *via* G protein signalling cascades. The first iGluR and mGluR members were cloned in 1989, and 1991 respectively and intense research into receptor subtypes and ligands has continued ever since in the search for possible new paradigms of treatment (22, 23).

Specific iGluR agonists have revealed three main subfamilies of receptors, named after the agonist: α-amino-3-hydroxy-5-methyl-4-isoxazolepropionic acid (AMPARs), kainic acid (KARs), and N-methyl-D-aspartate (NMDARs) (24). NMDARs, the target site of ketamine, were first cloned in 1991 (25). Within each receptor group genetic mechanisms generates further structural variations within the receptor subunits, which creates functional diversity (26). This receptor-encoded complexity sets glutamate apart from other neurotransmitters and enables the functional capacity required for complex processing in pattern and memory encoding.

Experimentally depression and anxiety like behaviours are reproduced by stimulating or blocking the subunits of these receptors with pharmacological ligands. Where specific receptor ligands are unknown, gene targeting models in mice can investigate the contribution of subunit subtypes. In a knockout mouse model of the GluN2A subunit of NMDARs, mice exhibited reduced anxiety and depression behaviour during experimental conditions such as the forced swim test and tail suspension tests (27). Similarly effects were seen with deletions of GluN1 GluN2B NMDA subunits, or the administration of the GluN2B antagonist Ro 25-6981 (28).

The precise mechanisms of NMDAR antagonism reducing depression are still unclear as pure NMDAR antagonists alone do not reduce depressive symptoms (29). The antidepressant effects of NMDAR antagonism in preclinical experiments require simultaneous activation of AMPARs (30). Although the reasons are unclear, the GluA1 subunit of AMPAR seems to be related to dysfunctional synaptic plasticity during depression. The selective deletion of this GluA1 AMPA subunit in the hippocampus, reduces experience dependent behavioural despair (31).

Glutamate is the predominant excitatory neurotransmitter in the central nervous system (CNS) accounting for over 90% of excitatory function (32). Glutamatergic synapses are found throughout the CNS but are especially concentrated in the hippocampus, amygdala, caudate nucleus, pre frontal cortex (PFC) and the cerebellum (33).

Glutamate neurone distribution therefore neatly overlays the main anatomical areas implicated in depression, namely the hippocampus and PFC. The excitatory nature of glutamate neurotransmission and vast array of receptor combinations, enables the complex synaptic activity required by the hippocampus for rapid pattern recognition. Additional functions include: spatial reference and working memory, pattern discernment and mapping, emotional activation and decision making, all in response to new environmental stimuli. Impairment of these functions leads to low mood and anhedonia. Prefrontal inhibition leads to inflexible negative biases in cognition, predisposing to rigidly held negative beliefs and poor motivation, leading to negative cognition arising the low mood (34).

It is the unique properties of glutamatergic neurotransmission, enabling memory and cognition, that create a vulnerability to dysfunction, leading to depression and other conditions. Unlike other neurotransmitters, glutamate causes synaptic excitation at micromolar concentrations and cannot then be enzymically deactivated in the synaptic space. Therefore, to avoid the lethal effects of synaptic overstimulation and excitotoxcity, adjacent astrocytes have developed a receptor-based system of reuptake transporters: GLT-1, GLAST, and EAAT (35). After uptake, glutamate is metabolised within the astrocyte, to non-excitatory glutamine and returned to the neurone *via* the glutamate–glutamine cycle, or oxidised and entered into the citric acid cycle as a fuel substrate. This active system of glutamate uptake against steep concentration gradients, requires significant amounts of ATP energy and therefore mitochondrial involvement. Glutamate neurotransmission therefore accounts for 80% of entire brain energy expenditure, and 20% of the total body expenditure. Hence, prolonged mental concentration, involving widespread glutamatergic activity, causes sensations of fatigue (36, 37).

The link of the glutamine-glutamate cycle with the tricarboxylic acid (TCA) cycle in astrocyte mitochondria, enables glutamate neurotransmission to recycle some of the cellular energy loss, thus avoiding potential local energy mismatch issues. Despite this, the net demands on astrocytic mitochondria remain very high and still render the hippocampus very vulnerable to metabolic overload or inflammatory stress. This dependency of hippocampal function and therefore mood, on mitochondrial function, is demonstrated by resveratrol, a drug whose action is purely to increase mitochondrial function. In animal models of depression, significant increases in mood are observed, without a direct effect on neurotransmitters (38).

Mitochondria, as providers of ATP energy for the cell, therefore also have a role in sensing and regulating cellular ATP requirements and oxidative stress. Mitochondria are therefore sensitive to numerous signalling pathways involving oxidative stress and inflammation. As such, the glutamate-based model of depression, through glutamatergic astrocytic mitochondria, demonstrates direct links between depression symptoms and physiological systems such as HPA stress responses, physical activity and less intuitively, the immune system.

Decades of depression mechanistic research have converged on three main, but interrelated, systems associated with depression; stress *via* the HPA axis, inflammation and neuroplasticity. This review discusses these aspects in relation to glutamate function, the hippocampus and physical activity.

## Glutamate mediates the relationship of stress with depression

The hippocampus, within the limbic system, is the primary centre of receiving and interpreting external stimuli and determining appropriate and rapid emotional, cognitive, neurological and endocrine responses. The primary endocrine response, when threats are perceived, is *via* the HPA axis, culminating in glucocorticoid release from the adrenal glands. The process starts when impulses from the hippocampus excite corticotrophin release factor (CRF) cells in the paraventricular nucleus (PVN) of the hypothalamus. The excitation is *via* glutamate receptors, demonstrating one of many relationships between glutamatergic activity and the endocrine stress response (39). Common to many glutaminergic microcircuits, GABA interneurones control the effect of excitation by inhibiting CRF cell secretion. GABA inhibition is itself deactivated when GABA ion channels open, causing depolarisation. Depolarisation is regulated by chloride cotransporters, which determine the transmembrane electrochemical gradient (40). In animal models, psychological stress and the inflammatory mediator IL-6 affect the number and function of these cotransporters and therefore act to deactivate GABA inhibition, hence increasing CRF and cortisol secretion (41). Cortisol acts *via* glucocorticoid (GR) receptors to cause GABA inhibition of CRF secretion and hippocampal deactivation to complete the negative feedback loop. Such close hippocampal-hypothalamic interactions explain the tight association of stress and mood.

In the short term, stress-induced deactivation of GABA, enabling cortisol-mediated protective mechanisms, is an appropriate physiological response to a perceived threat. Cortisol, the primary adrenal glucocorticoid, has anti-inflammatory and catabolic effects, necessary to contain infection, trauma and other defined periodic immune stresses.

Medium term stress, over weeks or months, such as bereavement, divorce, financial or criminal issues, and illness, produce perpetuation of the HPA axis activation. This occurs as a result of a self-amplification effect, whereby HPA glucocorticoids provide feedback to the CNS and activate GR receptors on the original glutamate presynaptic neurones, provoking further CRF cell activation. Although the underlying precipitants and perpetuating causes are not fully established, external agents that promote glutamatergic excitation and suppress GABA inhibition are clearly involved. This is sometimes referred to as “the GABA-ergic deficit hypothesis of major depressive disorder” (42). The high levels of circulating cortisol causes persistent GR receptor activation and a positive feedback loop.

GR receptor-stimulation also causes astrocytic release of ATP causing microglial proliferation, the release of pro-inflammatory mediators and the release of the growth factor Brain Derived Neurotrophic Factor (BDNF) (43). In animal models, the effects of persistent glucocorticoids on glutamate neurones are: increased glutaminergic excitability with astrocytic decline, reduced astroglial plasticity and reduced dendritic connectivity in hippocampal and frontal cortex regions (44, 45). Collectively this manifests as anxiety, depressed mood, disturbed or excessive sleep, fatigue, memory and cognitive problems, behavioural responses and, in extreme circumstances, suicide (46). Outside of the CN, cortisol causes depressed humoral and cellular immunity with dysmetabolic and vascular changes.

In short term stress, this cluster of cortisol-mediated, energy-conserving and self-protective symptoms, is seen in the “sickness behaviour” of people with severe infections. It also features behavioural changes, such as anorexia, fatigue, loss of interest in usual daily activities, social withdrawal, listlessness or malaise, hyperalgesia, sleep disturbances, and cognitive dysfunction (47). The “pathogen defence hypothesis of depression” is supported by the ancestral and continued proximity of alleles for depression and pro-inflammatory factors (48). This inflammatory depression of hippocampal function as an ancient protective mechanism becomes problematic in modern life when HPA activation is persistent.

The glutamate model explains why reduced physical activity, low motivation and depressed mood may in fact be inherent protective adaptations to stress and may explain the poor motivation and uptake of exercise in interventions for major depression.

From experimental simulations of stress, creating subtypes such as intermittent, restrained, or unpredictable forms, it is clear that high persistent cortisol levels also feature a pro-inflammatory state. This less understood aspect of the stress response is due to cortisol-mediated short term rises in plasma IL-6, TNF-alpha, and monocyte nuclear-factor kappa-B (NFkB), partially facilitated by the sympathetic nervous system (SNS) (49–51). As stress persists there is increasing SNS mediated B-adrenoceptor-activated monocyte migration into the CNS, causing pro-inflammatory cytokine secretion (52). Clearly, stress, anti-inflammatory HPA-stress responses, and raised pro-inflammatory mediators, have a complicated and sometimes seemingly contradictory relationship, although this may simply reflect different phases in the stress response and emphasises the dynamic nature of the hippocampal/HPA systems. This co-existent state of pro-inflammatory cytokines and raised CNS cortisol, causes double stimulation of astrocyte and neuronal receptors, leading to cellular exhaustion and apoptosis. This, in turn, leads to more chronic and structurally consolidated stress phenotypes (53).

## Prolonged stress

Continual suppression of GABA inhibition leads to glutamate overstimulation and eventual exhaustion of CRF cells and the HPA axis, resulting in low levels of glucocorticoids despite ongoing stress. Low circulating glucocorticoids are seen in atypical depression, post-traumatic stress disorder (PTSD) and in suicide attempts (54). Reduced circulating glucocorticoids remove the suppressive influence the HPA axis exerts on pro-inflammatory mediators. The resulting pro-inflammatory state causes dendritic damage, reduced synaptic plasticity and the transformation of astrocytes into pro-inflammatory phenotypes, which then secrete cytokines in a “feed-forward” manner. Pro-inflammatory cytokines then act to reduce mitochondrial biogenesis leading to metabolic insufficiency with autophagy and apoptosis.

In severe or prolonged stress, cumulative cell loss eventually results in loss of volume of hippocampal and prefrontal areas on MRI, with reductions in glutamate neurotransmission on MRS brain imaging, as described above. These macroscopic changes represent significant neuronal loss and degeneration and are commensurate with the increasing evidence of chronic MDD or other major psychiatric conditions, converting to neurodegenerative conditions such as Dementia and Parkinson’s Disease (55, 56). The transition from MDD to diseases featuring progressive neurological decline is unpredictable and poorly understood, but is consistent within the framework of the glutamatergic model.

Severe stress experienced in early life seems to amplify pro-inflammatory responses to stress in adulthood, possibly as a result of defective HPA/hippocampal integration during development, although the mechanism is not fully understood (57). The resulting chronic pro-inflammatory states may explain the connection between adverse life events in childhood and subsequent depression and chronic physical disease. Childhood Adverse Life Experiences (ALEs) are associated with a 2.4-times increase in mortality at the age of 65, compared to controls (58).

## Glutamate, inflammation and depression

Patients with major depression have significantly higher plasma concentrations of the cytokines TNF-α and IL-6 than controls (59). Whilst CNS inflammatory mediators form part of the depression-associated stress response above, primary inflammatory conditions themselves can invoke depression (60). The relationship between inflammation and depression therefore is rather circular. Regardless of whether the origin is centrally derived or peripheral, inflammatory mediators produce similar effects on glutamate microcircuits to those seen with stress, namely increased oxidative and nitrosative stress, with reduced BDNF and other growth factors needed for maintenance of the glutamate astro-neuronal unit. Oxidative stress results from the imbalance of between mitochondrial reactive oxygen species ROS production and removal and therefore excess superoxide and other ROS within the cell. This arises from overproduction and or deceased antioxidant capacity. Oxidative damage affects many cellular components, including lipids DNA and proteins and mitochondria themselves. Up to 2% of consumed oxygen is converted to superoxide through electron leakage within the mitochondrial ETC (61). In energetic cells, such as neurons, with raised mitochondrial activity, ROS production is increased (62). However ROS production is also increased when mitochondria become impaired and electron leakage increases (63).

In addition to the direct effects on cells, ROS also stimulates pro-inflammatory cytokines such as IL-6 and TNFa. This provokes a ligand-activated transcription factor: peroxisome-proliferator-activated receptor-γ (PPAR-γ). PPAR-γ suppresses pro-inflammatory transcription factors and activates mitochondrial biogenesis. This key metabolic controller also promotes anti-oxidant and angiogenesis pathways and raises glucose and lactate availability in astrocytes (64).

The high oxidative activity of neuronal mitochondria, makes the mitochondria ETC itself vulnerable to functional overload, producing excess ROS, ETC damage, then increased electron leakage and ROS in a vicious circle. Mitochondrial function declines and antioxidant defences are reduced. The increased oxidative state damages organelles and if prolonged risks progression to autophagy, *via* mTor signalling and apoptosis *via* activated Caspases (65, 66).

## Effects of physical activity in increasing neuronal mitochondrial biogenesis

Counterbalancing these risks of functional overload are trophic mechanisms to promote cell enhancement and growth, which are strongly related to physical activity. PGC1a is a transcriptional co-activator stimulated by physical activity which, *via* PPAR-γ, facilitates mitochondrial biogenesis (67). This effect is not just confined to muscle. In the hippocampus, exercise induced biogenesis increases levels of oxidative phosphorylation giving neuroprotection to glutamate neurones, vulnerable to mitochondrial insufficiency (68). PGC1a is activated in muscle by a mitochondrial energy-sensing kinase, AMPK and stress inducible kinases including p38 mitogen-activated protein kinase (MAPK). Muscle activity in the form of exercise, particularly endurance exercise, creates energy demand and optimally stimulate production, with effects in the CNS (69).

In patients with treatment resistant depression (TRD), that is not responsive to serotoninergic drugs, it would be expected that exercise would stimulate increased PGC1a and hence hippocampal mitogenesis to reduce pro-inflammatory cytokines and improve mood. This was in fact observed in a study of 12 weeks of an aerobic exercise protocol in TRD patients (70). Increased mood correlated with reduction of IL-1β levels. Notably the higher the circulating basal TNF-α levels the greater the effects of PA on reducing the depression.

Physical activity also serves to lose weight, particularly visceral adiposity, which coupled with sedentariness are potent contributors of circulatory IL-6 and TNF-α, sometimes described as the “diseaseome of physical inactivity” (71).

Randomised trials show positive results for exercise improving mental health but due to the heterogeneity in responses, effect sizes are smaller than in large observational studies (72).

Most evidence indicates that exercise, especially aerobic exercise improves depression and anxiety outcome measures, with similar effect sizes to psychopharmacological treatments. Resistance exercise trials also show reduction in symptoms (73). Exercise benefits are seen in different forms of depression such as in the elderly and children and young people (74, 75). Notably the mental health effects continue during the intervention but decline rapidly after discontinuation (76). Sustained beneficial effects seem to be maintained only above a threshold of 150 mins per week, as reflected by WHO exercise guidelines (77). The considerable variation of exercise effects across patient populations and forms of activity indicates the need to understand the underlying mechanisms, although this has proved elusive. The discovery of the kynurenine pathway, linking muscle activity with neurophysiology was a major development in understanding.

## Physical activity and the kynurenine pathway

A key inflammatory pathway in CNS glutamatergic systems, relevant to PA, is the kynurenine pathway. The kynurenines are bioactive metabolites of tryptophan (TRP), deriving from the gut and are therefore an important feature of traffic in the gut-muscle-brain axis (78). Much interest has focused on members of this pathway in terms of mediating inflammatory CNS conditions. In the brain kynurenine metabolites are generated locally by microglia or enter *via* the blood-brain barrier (BBB) from the circulation. Importantly, excess kynurenine substrate (KYN) is prevented from entering the brain, through conversion by exercising muscle into kynurenic acid (KA), which cannot pass the BBB. In the brain, kynurenine catabolism divides into two pathways depending on local conditions: Either pro-inflammatory neurotoxic metabolites including Quinolinic acid, (QA) (a glutamate NMDA receptor agonist), or neuroprotective metabolites including kynurenic acid, KynA (an NMDAr antagonist). Initially, the kynurenine pathway was thought to cause depression by depleting serotonin, but the principal site of action is now known to be glutamatergic transmission (79). The ratio of KynA to QuinA has been demonstrated to be low (i.e., neurotoxic), due to the excess QuinA released from microglial cells, in the anterior cingulate cortex in depression (80). Moreover, the kynurenine pathway itself is modulated peripherally in adverse conditions such as infection and stress. However, in exercise, beneficial effects occur, with muscle enzyme increases or decreases, causing changes in the KynA:QuinA ratio (81). In addition to reducing CNS entry of kynurenine, exercise promotes enzymic switches towards an increased KynA/QuinA ratio, which promotes PGC1a mediated mitochondrial biogenesis. This is responsible for the beneficial effects of exercise such as improved energy homeostasis, the promotion of an anti-inflammatory environment, and neuroprotection, and is thought to be one of the mechanisms underlying the beneficial effects of exercise on depression (82).

## Neuroplasia and depression

From a structural point of view, the hippocampus is a central processor of incoming information, rapidly sorting, evaluating and storing sensory input and connecting with the PFC, amygdala and HPA axis for cognitive, emotional and endocrine responses. The speed and complexity of this most challenging of brain activities requires high levels of neurogenesis and neuroplasticity in these exclusively glutamate neurones. Neurogenesis, the formation of new neurone cells, continues throughout adult life, only in two areas of the adult brain; a zone of the lateral ventricles and the dentate gyrus of the hippocampus (83). Neurogenesis seems a prerequisite for memory formation. Neuroplasticity involving the growth, consolidation and pruning of hippocampal dendrites is a vital part of memory consolidation and memory removal, when modelled by activity-dependent changes in synaptic transmission such as long-term potentiation (LTP) and long-term depression (LTD) (84). Stress acts through many mechanisms to impair synaptic plasticity and neurogenesis, by direct decrease of dendritic branching or activating corticosteroids to downregulate neurogenesis (85). Severe and continued impairment is reflected in overall loss of hippocampal volume.

As described above, the link between impairment in the memory forming regions of the hippocampus and depression is strong but not self-evident. However evidence is incontrovertible that reduced neuroplasticity in areas of memory function correlates to symptoms of depression (86). Trophic factors that promote synaptogenesis or improve mitochondrial function to fuel the process therefore also improve mood. Growth factors, or neurotrophins, are produced locally or enter *via* the BBB from muscle and other tissues. The mammalian neurotrophins include: Brain derived neurotrophic factor (BDNF), insulin like growth factor (IGF-1) vascular endothelial growth factor (VEGF) fibroblast growth factor (FGF-2) epidermal growth factor (EGF) nerve growth factor (NGF) (87). Irisin, produced in muscle, is involved in the regulation of neural differentiation, neuroplasticity and energy expenditure (88).

BDNF is a vital growth factor for sustaining neurones and promoting growth and repair and is synthesised in neurones in physiological circumstances and astrocytes during inflammation or injury (89). BDNF expression is highest in hippocampal and frontal areas corresponding to emotional and cognitive function. It is well established that serum BDNF levels are reduced in depression and are normalised with treatment by antidepressants or PA (90). PA increases plasma levels threefold, partially due to muscle derived BDNF and contributes to brain plasticity and neurogenesis. Aerobic and anaerobic exercises promote neurogenesis *via* BDNF, lactate and Vascular Endothelial Growth Factors (VEGF). In contrast, for resistance exercise, neuroplasticity and neurogenesis is mediated *via* muscle derived irisin and IGF-1 (91).

As BDNF expression features highly in those hippocampal circuits responsible for interpreting external sensory information, then environmental enrichment appears to increase BDNF further and be the primary source of the ability of environmental enrichments to enhance cognitive processes (92).

## Summary of neurological effects of PA

**Figure 1:**
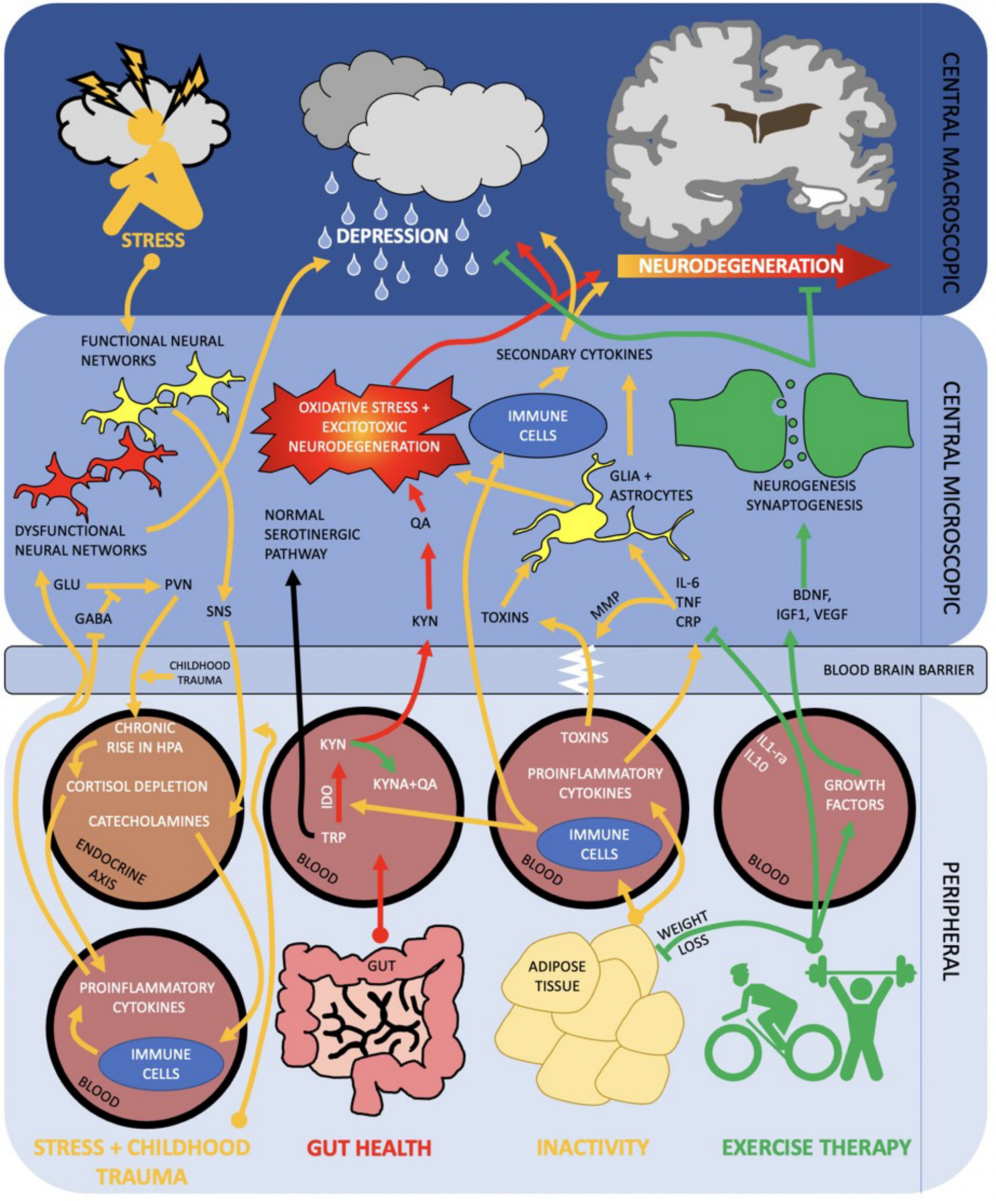
Diagram of the brain and peripheral neuroimmune interactions in terms of the gut—muscle-brain axes. RED activation of kynurenine pathway *via* poor gut health propagates oxidative stress and excitotoxic neurodegeneration. ORANGE inactivity and subsequent raised adiposity elicits an inflammatory cascade which traverses the blood brain barrier and contributes to neurodegeneration and depression. Stress and negative early life events may activate a parallel pathway, in which peripheral inflammation and HPA axis dysregulation contribute to dysfunctional neural networks and depression. GREEN exercise therapy both acutely (arrow) and chronically (inhibition arrow) inhibit inflammatory and kynurenine pathways whilst promoting neurogenesis and synaptogenesis. Combined, this can help stave off neurodegeneration and onset of depression. SNS, sympathetic nervous system; MMP, mixed metalloproteases; PV, paraventricular nucleus; IDO, indoleamine 2,3-dioxygenase; Other abbreviations described above.

Physical activity significantly improves brain function as measured by neuronal survival, resilience to brain physical insults, cognitive function, brain vascularisation, neuroplasticity and neurogenesis, neuroinflammatory stability and resistance to the effects of ageing. Physical activity mechanisms operate not in isolation but form part of the network of the gut-muscle-brain axes, in addition to environmental stressors as depicted in Figure 1.

The primary focus of these effects centres on the hippocampus and involves glutamatergic neurotransmission. Key to maintaining homeostasis and promoting growth are the neurotrophic factors produced locally or peripherally in muscle, which emphasises the crosstalk between brain and the musculoskeletal system. In terms of neuropsychiatric conditions, these changes have the effect of stabilising mood, reducing negative cognitive bias, increasing resilience to psychosocial stress and increasing motivation (93). There is also evidence of exercise directly stimulating hippocampal circuitry from other parts of the CNS. Glutamate neurotransmission frequencies are seen to increase almost instantaneously to the start of exercise. The mechanisms and neural pathways are unclear but the neuroplastic promoting effects on hippocampal neuronal health are similar to the other effects of PA (94).

In addition to the direct neurohumoral benefits of exercise, PA also provides numerous indirect benefits due to the nature of different sports and the external environment. Complex tasks, such as strategizing and decision making, risk management and social interaction, all stress and stimulate hippocampal and PFC circuits in beneficial ways (95). Exercise tends to behaviourally influence other positive activities such as diet quality and social interactions, all with individual beneficial effects on neuroimmunology (96).

In a negative context, the importance of environment is illustrated by the increasing incidence of mental health disorders in professional athletes, illustrating the power of environmental stress factors to override the antidepressant effects of even large quantities of physical activity (97).

## PA in other neuropsychiatric conditions

The physiological effects of exercise impact positively on the vast majority of psychiatric conditions from affective disorders, schizophrenia disorders to trauma-based and developmental disorders.

Unlike the glutamate model, many of the previous depression models of pathogenesis do not transfer across diagnostic boundaries. In a previous review we described the validity of the glutamatergic model across diverse neuropsychiatric categories, involving common themes such as excitotoxicity, and suggest possible novel treatment avenues (98). Through cytokines and neurotrophins the glutamate model is also able to provide a theoretical basis for physical activity impacting on depression and other psychiatric disorders. Many disorders that feature depression and hippocampal pathology, respond to PA as described. However, other disorders may improve along different channels and await further research. A full description of the clinical research on the benefits of physical activity on the spectrum of neuropsychiatric conditions is outside the scope of this mechanism-based review. However, Table 1 summarises the evidence of effect, taken from systematic reviews, where available, in the main DSM-5 disease categories.

**Table 1:** Quantity of evidence for physical activity influencing neuropsychiatric conditions (Symbols “+”, “++”, “+++” and “++++” refer to approximate proportional quantities, relative to zero).

DSM V classification	Evidence of PA preventing disease	Evidence of PA treating symptoms	Volume of evidence
Depressive disorders	+++	+++	++++
Anxiety	+++	+++	++++
Schizophrenia	0	++	(99, 100)
Bipolar disorder	0	++	Paucity (101)
**Neurodevelopmental disorders**			
Autism	0	+	+ (102)
ADHD	0	+	+ (103)
Intellectual disorders	0	+	+ (104)
**Trauma and stressor related disorders**			
PTSD	0	++	+ (105)
OCD	0	+	+ (106, 107)
Personality disorders	0	0	No studies
**Neurodegenerative disorders**			
Alzheimers	+++ (108)	++ (109)	++
Parkinsons	+++ (110)	+++ (111)	+++

It is interesting to note the contemporaneous nature of much of this research, which demonstrates how psychopharmacology has dominated psychiatric treatment research in previous decades. Also, the inconsistency in the volume of research for each disorder, across the spectrum, is evident in Table 1. While there is a substantial research base into PA for depression and neurodegenerative disorders, research into bipolar disorder and schizophrenia is a more recent phenomenon and much smaller in volume. Research into clinical benefits in the remaining categories is at an embryonic stage, despite strong evidence of underlying glutamatergic dysfunction and therefore plausible responses to PA. At the time of writing there are no identifiable studies of PA in Personality Disorders. The reasons for this are not clear.

## The seesaw model of hippocampal glutamatergic function

Multiple previous models of depression (inflammatory, neuroplastic) have been discussed in relation to increasing evidence supporting glutamate neurotransmission as the key player. Many previous theories consider systems in isolation and without temporal features. As a result most theories, although partially correct, struggle to incorporate or explain the complex effects of exercise on depression neurobiology.

**Figure 2:**
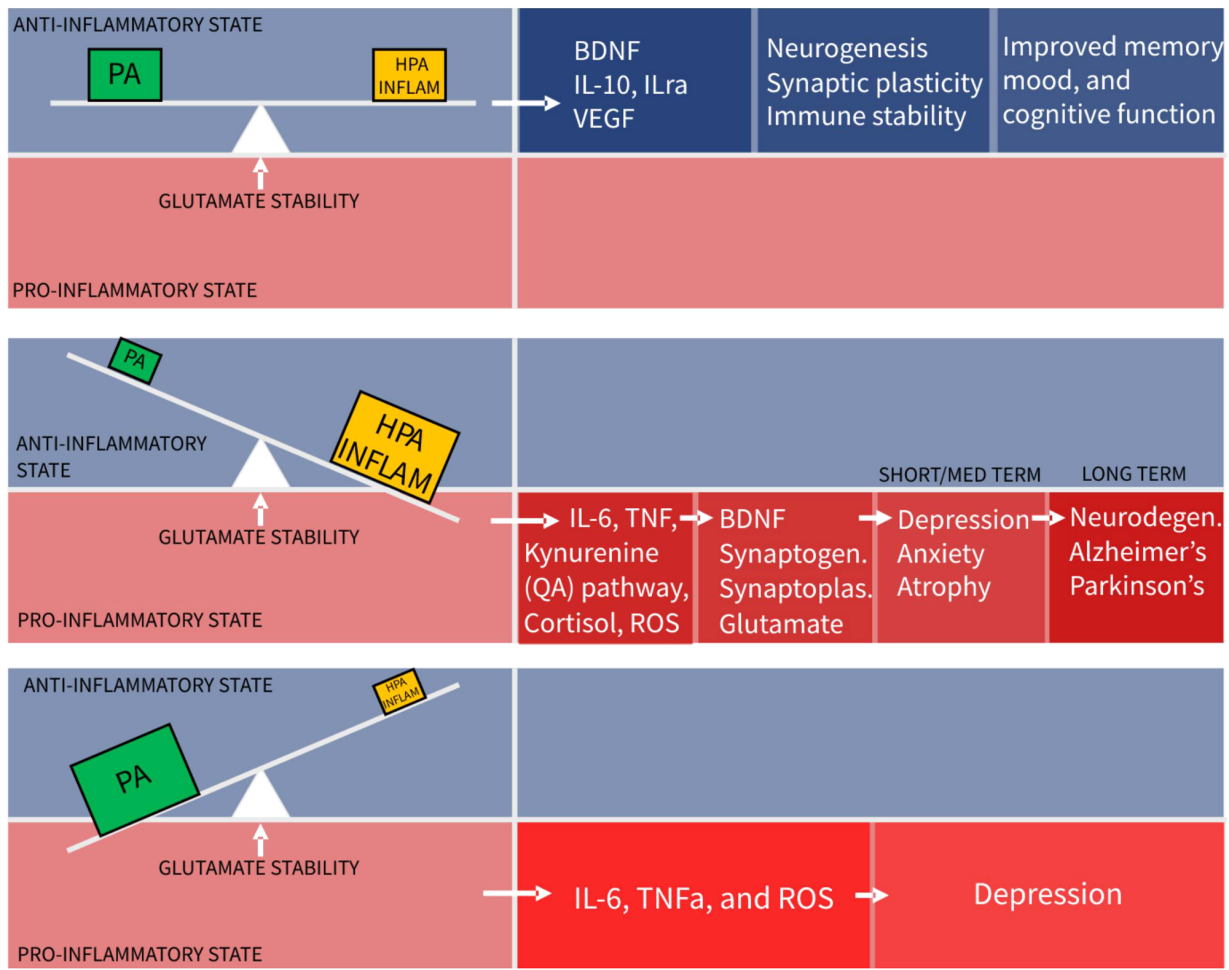
The seesaw principle of glutamatergic homeostasis (PA, physical activity; HPA, hypothalamo-pituitary-adrenal axis).

The conclusion from this review is that physical activity, HPA stress, and neuroinflammation are three distinct, necessary and constant influences forming a dynamical balance or “seesaw”, as depicted in Figure 2, that controls glutamate neurotransmission in the hippocampus and PFC, to maintain mood and cognitive function. Numerous other factors such as diet and genetic influences are relevant but for simplification only the best known influencers of glutamatergic stability are included.

It is the hope that providing a robust simple model will enable researchers to identity gaps and conduct much needed research in this field.

The implication of this model is that physical activity is not an adjunctive treatment for depression, but an essential component of basic functioning, without which harm is caused. This absence of PA, (sedentariness), is predicted this model to tip the balance towards a pro-inflammatory state. Indeed chronic sedentariness is seen to correlate with chronic low-grade pro-inflammatory states leading to depression and cardiometabolic disease.

## Too much of a good thing

A second implication is that moderate amounts of hippocampal stimulation from the HPA axis and neuroinflammation systems are required for system stability, despite often being considered as pathological forces. With dynamic systems the quantity and temporal behaviour sometimes determine the beneficial or harmful nature of the agent. Taking this to an extreme, to demonstrate proof of model concept, it can be seen that even beneficial physical activity, in excessive amounts, can cause a pro- inflammatory state and be harmful. Excessive exercise training schedules with insufficient recovery time and extreme ultra-distance running events causing muscle injury and pro-inflammatory states are common examples of this (112–114).

## Too little of a bad thing

The converse scenario of the seesaw model shows too little stimulation from HPA or inflammatory systems. This leads to reduced levels of BDNF, reduced synaptoplasticity and a slow decline in glutamate functionality. Clinically there is evidence of anxiety and depression resulting from lack of stress and stimulation (115). Social isolation is related to this. Small amounts of “healthy” acute stress such as public speaking, produce cortisol mediated dentate gyrus neurogenesis leading to improvements in memory and performance 2 weeks after stress exposure (116).

How much physical activity is required to maintain health or homeostatic balance, is seen in this model to be a varying and dynamic quantity, depending on the counterbalancing stress and inflammation within the individual. Hence, increased stress, tipping glutamate neurones towards dysfunction can be rebalanced by increased amounts of physical activity and this is borne out in clinical research. The underlying state of individual balances may explain some of the variation in responses of individuals to mass exercise interventions for mental health and the difficulties in prescribing optimal exercise interventions for individuals.

Another application of this model is in the depression associated with the chronic metabolic diseases, such as diabetes, characterised by low grade pro-inflammatory states. In this case, systemic cytokines enter the brain causing neuroinflammation with reduced mood and cognition. This condition has only relatively recently been recognised despite affecting up to 20% of patients (117).

As a result there is very little research available to date, on PA interventions to improve this form of depression in chronic disease groups. Limited studies have shown improvement in mood and motivation in chronic lung disease, diabetes and rheumatoid patients after exercise, independent of the state of the underlying condition (118).

The important implication of the glutamate model is that treatment of chronic disease-associated depression, by physical activity, not only reduces depression symptoms in current disease, but is neuroprotective against future chronic metabolic disease-associated-neurodegeneration, such as diabetes associated dementia and Parkinson’s disease. This is also a recently recognised and significant clinical burden, which is not currently addressed by conventional disease management (119, 120).

In a parallel process, the glutamate balance model also indicates that chronic psychiatric conditions, such as Major Depressive Disorder, could theoretically progress on to later neurodegenerative disease. Conditions where PA is less studied, such as Borderline Personality Disorder, theoretically risk future neurodegenerative disease, because they already demonstrate the pre-requisites of chronic inflammation and reduced hippocampal volume as a result of developmental in HPA responses (121). This emphasises the urgent need for neuroprotective exercise research in these poorly studied groups and other psychiatric conditions that stem from Adverse Childhood Experiences (ACEs).

Lastly it is important to distinguish this dynamic glutamate “seesaw” model based on the non-linear laws of cellular energetics and energy balance, from the psychological associations observed in depression. Hence stress and depression are often associated with reduced physical activity levels, despite the fact that this model clearly indicates the need to increase. The psychology of why human behaviour does not necessarily follow what the underlying balance state is indicating, is complex and outside the subject of this review.
